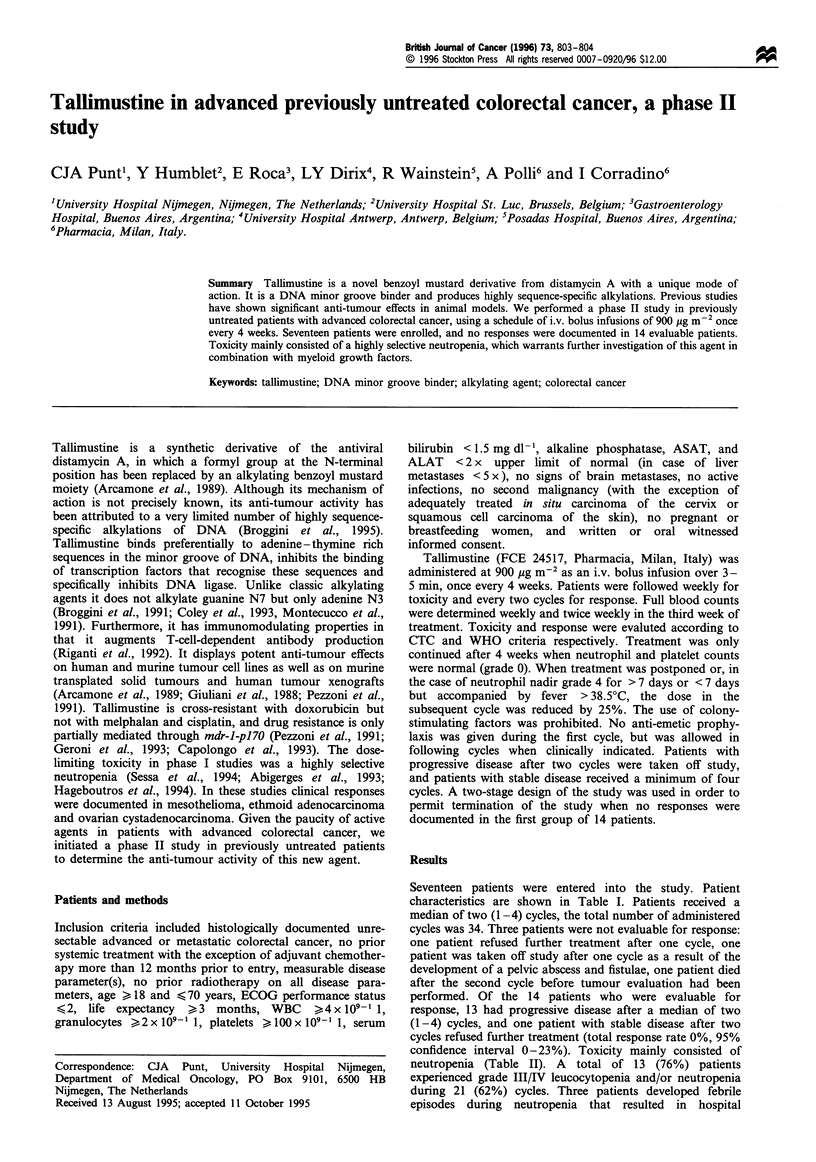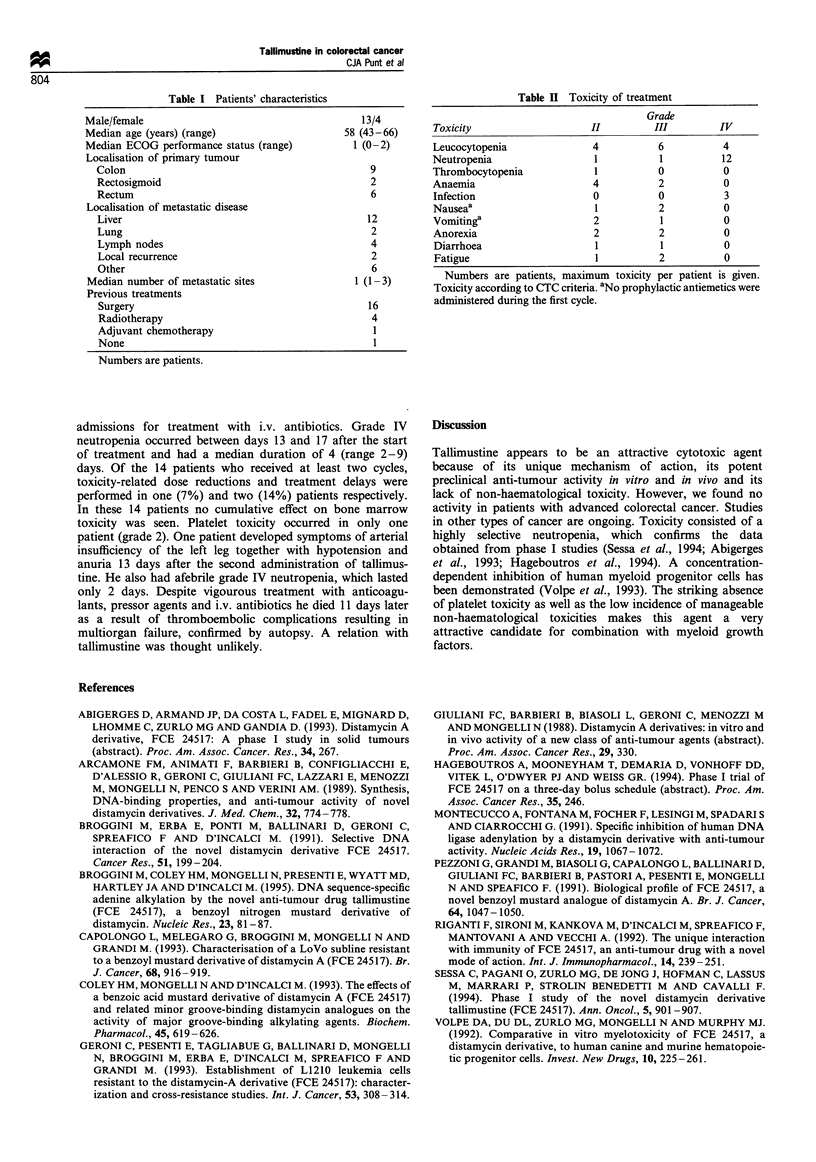# Tallimustine in advanced previously untreated colorectal cancer, a phase II study.

**DOI:** 10.1038/bjc.1996.140

**Published:** 1996-03

**Authors:** C. J. Punt, Y. Humblet, E. Roca, L. Y. Dirix, R. Wainstein, A. Polli, I. Corradino

**Affiliations:** University Hospital Nijmegen, The Netherlands.

## Abstract

Tallimustine is a novel benzoyl mustard derivative from distamycin A with a unique mode of action. It is a DNA minor groove binder and produces highly sequence-specific alkylations. Previous studies have shown significant anti-tumour effects in animal models. We performed a phase II study in previously untreated patients with advanced colorectal cancer, using a schedule of i.v. bolus infusions of 900 microgram m-2 once every 4 weeks. Seventeen patients were enrolled, and no responses were documented in 14 evaluable patients. Toxicity mainly consisted a highly selective neutropenia, which warrants further investigation of this agent in combination with myeloid growth factors.


British Journal of Cancer (1996) 73, 803-804
© 1996 Stockton Press All rights reserved 0007-0920/96 $12.00

### Tallimustine in advanced previously untreated colorectal cancer, a phase II study

CJA Punt[1], Y Humblet[2], E Roca[3], LY Dirix[4], R Wainstein[5], A Polli[6] and I Corradino[6]

[1]*University Hospital Nijmegen, Nijmegen, The Netherlands;* [2]*University Hospital St. Luc, Brussels, Belgium;* [3]*Gastroenterology Hospital, Buenos Aires, Argentina;* [4]*University Hospital Antwerp, Antwerp, Belgium;* [5]*Posadas Hospital, Buenos Aires, Argentina;* [6]*Pharmacia, Milan, Italy.*

**Summary** Tallimustine is a novel benzoyl mustard derivative from distamycin A with a unique mode of action. It is a DNA minor groove binder and produces highly sequence-specific alkylations. Previous studies have shown significant anti-tumour effects in animal models. We performed a phase II study in previously untreated patients with advanced colorectal cancer, using a schedule of i.v. bolus infusions of 900 $\mu$g m$^{-2}$ once every 4 weeks. Seventeen patients were enrolled, and no responses were documented in 14 evaluable patients. Toxicity mainly consisted of a highly selective neutropenia, which warrants further investigation of this agent in combination with myeloid growth factors.

**Keywords:** tallimustine; DNA minor groove binder; alkylating agent; colorectal cancer

Tallimustine is a synthetic derivative of the antiviral distamycin A, in which a formyl group at the N-terminal position has been replaced by an alkylating benzoyl mustard moiety (Arcamone *et al.*, 1989). Although its mechanism of action is not precisely known, its anti-tumour activity has been attributed to a very limited number of highly sequence-specific alkylations of DNA (Broggini *et al.*, 1995). Tallimustine binds preferentially to adenine–thymine rich sequences in the minor groove of DNA, inhibits the binding of transcription factors that recognise these sequences and specifically inhibits DNA ligase. Unlike classic alkylating agents it does not alkylate guanine N7 but only adenine N3 (Broggini *et al.*, 1991; Coley *et al.*, 1993, Montecucco *et al.*, 1991). Furthermore, it has immunomodulating properties in that it augments T-cell-dependent antibody production (Riganti *et al.*, 1992). It displays potent anti-tumour effects on human and murine tumour cell lines as well as on murine transplated solid tumours and human tumour xenografts (Arcamone *et al.*, 1989; Giuliani *et al.*, 1988; Pezzoni *et al.*, 1991). Tallimustine is cross-resistant with doxorubicin but not with melphalan and cisplatin, and drug resistance is only partially mediated through *mdr-1-p170* (Pezzoni *et al.*, 1991; Geroni *et al.*, 1993; Capolongo *et al.*, 1993). The dose-limiting toxicity in phase I studies was a highly selective neutropenia (Sessa *et al.*, 1994; Abigerges *et al.*, 1993; Hageboutros *et al.*, 1994). In these studies clinical responses were documented in mesothelioma, ethmoid adenocarcinoma and ovarian cystadenocarcinoma. Given the paucity of active agents in patients with advanced colorectal cancer, we initiated a phase II study in previously untreated patients to determine the anti-tumour activity of this new agent.

### Patients and methods

Inclusion criteria included histologically documented unresectable advanced or metastatic colorectal cancer, no prior systemic treatment with the exception of adjuvant chemotherapy more than 12 months prior to entry, measurable disease parameter(s), no prior radiotherapy on all disease parameters, age $\geqslant 18$ and $\leqslant 70$ years, ECOG performance status $\leqslant 2$, life expectancy $\geqslant 3$ months, WBC $\geqslant 4\times10^{9-1}$ 1, granulocytes $\geqslant 2\times10^{9-1}$ 1, platelets $\geqslant 100\times10^{9-1}$ 1, serum bilirubin $<1.5$ mg dl$^{-1}$, alkaline phosphatase, ASAT, and ALAT $<2\times$ upper limit of normal (in case of liver metastases $<5\times$), no signs of brain metastases, no active infections, no second malignancy (with the exception of adequately treated *in situ* carcinoma of the cervix or squamous cell carcinoma of the skin), no pregnant or breastfeeding women, and written or oral witnessed informed consent.

Tallimustine (FCE 24517, Pharmacia, Milan, Italy) was administered at 900 $\mu$g m$^{-2}$ as an i.v. bolus infusion over 3–5 min, once every 4 weeks. Patients were followed weekly for toxicity and every two cycles for response. Full blood counts were determined weekly and twice weekly in the third week of treatment. Toxicity and response were evaluted according to CTC and WHO criteria respectively. Treatment was only continued after 4 weeks when neutrophil and platelet counts were normal (grade 0). When treatment was postponed or, in the case of neutrophil nadir grade 4 for $>7$ days or $<7$ days but accompanied by fever $>38.5$°C, the dose in the subsequent cycle was reduced by 25%. The use of colony-stimulating factors was prohibited. No anti-emetic prophylaxis was given during the first cycle, but was allowed in following cycles when clinically indicated. Patients with progressive disease after two cycles were taken off study, and patients with stable disease received a minimum of four cycles. A two-stage design of the study was used in order to permit termination of the study when no responses were documented in the first group of 14 patients.

### Results

Seventeen patients were entered into the study. Patient characteristics are shown in Table I. Patients received a median of two (1–4) cycles, the total number of administered cycles was 34. Three patients were not evaluable for response: one patient refused further treatment after one cycle, one patient was taken off study after one cycle as a result of the development of a pelvic abscess and fistulae, one patient died after the second cycle before tumour evaluation had been performed. Of the 14 patients who were evaluable for response, 13 had progressive disease after a median of two (1–4) cycles, and one patient with stable disease after two cycles refused further treatment (total response rate 0%, 95% confidence interval 0–23%). Toxicity mainly consisted of neutropenia (Table II). A total of 13 (76%) patients experienced grade III/IV leucocytopenia and/or neutropenia during 21 (62%) cycles. Three patients developed febrile episodes during neutropenia that resulted in hospital

Correspondence: CJA Punt, University Hospital Nijmegen, Department of Medical Oncology, PO Box 9101, 6500 HB Nijmegen, The Netherlands
Received 13 August 1995; accepted 11 October 1995

**Table I** Patients' characteristics

| | |
|---|---|
| Male/female | 13/4 |
| Median age (years) (range) | 58 (43-66) |
| Median ECOG performance status (range) | 1 (0-2) |
| Localisation of primary tumour | |
| Colon | 9 |
| Rectosigmoid | 2 |
| Rectum | 6 |
| Localisation of metastatic disease | |
| Liver | 12 |
| Lung | 2 |
| Lymph nodes | 4 |
| Local recurrence | 2 |
| Other | 6 |
| Median number of metastatic sites | 1 (1-3) |
| Previous treatments | |
| Surgery | 16 |
| Radiotherapy | 4 |
| Adjuvant chemotherapy | 1 |
| None | 1 |

Numbers are patients.

**Table II** Toxicity of treatment

| *Toxicity* | *Grade* *II* | *III* | *IV* |
|---|---|---|---|
| Leucocytopenia | 4 | 6 | 4 |
| Neutropenia | 1 | 1 | 12 |
| Thrombocytopenia | 1 | 0 | 0 |
| Anaemia | 4 | 2 | 0 |
| Infection | 0 | 0 | 3 |
| Nausea[a] | 1 | 2 | 0 |
| Vomiting[a] | 2 | 1 | 0 |
| Anorexia | 2 | 2 | 0 |
| Diarrhoea | 1 | 1 | 0 |
| Fatigue | 1 | 2 | 0 |

Numbers are patients, maximum toxicity per patient is given. Toxicity according to CTC criteria. [a]No prophylactic antiemetics were administered during the first cycle.

admissions for treatment with i.v. antibiotics. Grade IV neutropenia occurred between days 13 and 17 after the start of treatment and had a median duration of 4 (range 2-9) days. Of the 14 patients who received at least two cycles, toxicity-related dose reductions and treatment delays were performed in one (7%) and two (14%) patients respectively. In these 14 patients no cumulative effect on bone marrow toxicity was seen. Platelet toxicity occurred in only one patient (grade 2). One patient developed symptoms of arterial insufficiency of the left leg together with hypotension and anuria 13 days after the second administration of tallimustine. He also had afebrile grade IV neutropenia, which lasted only 2 days. Despite vigourous treatment with anticoagulants, pressor agents and i.v. antibiotics he died 11 days later as a result of thromboembolic complications resulting in multiorgan failure, confirmed by autopsy. A relation with tallimustine was thought unlikely.

### Discussion

Tallimustine appears to be an attractive cytotoxic agent because of its unique mechanism of action, its potent preclinical anti-tumour activity *in vitro* and *in vivo* and its lack of non-haematological toxicity. However, we found no activity in patients with advanced colorectal cancer. Studies in other types of cancer are ongoing. Toxicity consisted of a highly selective neutropenia, which confirms the data obtained from phase I studies (Sessa *et al.*, 1994; Abigerges *et al.*, 1993; Hageboutros *et al.*, 1994). A concentration-dependent inhibition of human myeloid progenitor cells has been demonstrated (Volpe *et al.*, 1993). The striking absence of platelet toxicity as well as the low incidence of manageable non-haematological toxicities makes this agent a very attractive candidate for combination with myeloid growth factors.

### References

ABIGERGES D, ARMAND JP, DA COSTA L, FADEL E, MIGNARD D, LHOMME C, ZURLO MG AND GANDIA D. (1993). Distamycin A derivative, FCE 24517: A phase I study in solid tumours (abstract). *Proc. Am. Assoc. Cancer. Res.*, **34**, 267.

ARCAMONE FM, ANIMATI F, BARBIERI B, CONFIGLIACCHI E, D'ALESSIO R, GERONI C, GIULIANI FC, LAZZARI E, MENOZZI M, MONGELLI N, PENCO S AND VERINI AM. (1989). Synthesis, DNA-binding properties, and anti-tumour activity of novel distamycin derivatives. *J. Med. Chem.*, **32,** 774-778.

BROGGINI M, ERBA E, PONTI M, BALLINARI D, GERONI C, SPREAFICO F AND D'INCALCI M. (1991). Selective DNA interaction of the novel distamycin derivative FCE 24517. *Cancer Res.*, **51,** 199-204.

BROGGINI M, COLEY HM, MONGELLI N, PRESENTI E, WYATT MD, HARTLEY JA AND D'INCALCI M. (1995). DNA sequence-specific adenine alkylation by the novel anti-tumour drug tallimustine (FCE 24517), a benzoyl nitrogen mustard derivative of distamycin. *Nucleic Res.*, **23,** 81-87.

CAPOLONGO L, MELEGARO G, BROGGINI M, MONGELLI N AND GRANDI M. (1993). Characterisation of a LoVo subline resistant to a benzoyl mustard derivative of distamycin A (FCE 24517). *Br. J. Cancer*, **68,** 916-919.

COLEY HM, MONGELLI N AND D'INCALCI M. (1993). The effects of a benzoic acid mustard derivative of distamycin A (FCE 24517) and related minor groove-binding distamycin analogues on the activity of major groove-binding alkylating agents. *Biochem. Pharmacol.*, **45,** 619-626.

GERONI C, PESENTI E, TAGLIABUE G, BALLINARI D, MONGELLI N, BROGGINI M, ERBA E, D'INCALCI M, SPREAFICO F AND GRANDI M. (1993). Establishment of L1210 leukemia cells resistant to the distamycin-A derivative (FCE 24517): characterization and cross-resistance studies. *Int. J. Cancer*, **53,** 308-314.

GIULIANI FC, BARBIERI B, BIASOLI L, GERONI C, MENOZZI M AND MONGELLI N (1988). Distamycin A derivatives: in vitro and in vivo activity of a new class of anti-tumour agents (abstract). *Proc. Am. Assoc. Cancer Res.*, **29,** 330.

HAGEBOUTROS A, MOONEYHAM T, DEMARIA D, VONHOFF DD, VITEK L, O'DWYER PJ AND WEISS GR. (1994). Phase I trial of FCE 24517 on a three-day bolus schedule (abstract). *Proc. Am. Assoc. Cancer Res.*, **35,** 246.

MONTECUCCO A, FONTANA M, FOCHER F, LESINGI M, SPADARI S AND CIARROCCHI G. (1991). Specific inhibition of human DNA ligase adenylation by a distamycin derivative with anti-tumour activity. *Nucleic Acids Res.*, **19,** 1067-1072.

PEZZONI G, GRANDI M, BIASOLI G, CAPALONGO L, BALLINARI D, GIULIANI FC, BARBIERI B, PASTORI A, PESENTI E, MONGELLI N AND SPEAFICO F. (1991). Biological profile of FCE 24517, a novel benzoyl mustard analogue of distamycin A. *Br. J. Cancer*, **64,** 1047-1050.

RIGANTI F, SIRONI M, KANKOVA M, D'INCALCI M, SPREAFICO F, MANTOVANI A AND VECCHI A. (1992). The unique interaction with immunity of FCE 24517, an anti-tumour drug with a novel mode of action. *Int. J. Immunopharmacol.*, **14,** 239-251.

SESSA C, PAGANI O, ZURLO MG, DE JONG J, HOFMAN C, LASSUS M, MARRARI P, STROLIN BENEDETTI M AND CAVALLI F. (1994). Phase I study of the novel distamycin derivative tallimustine (FCE 24517). *Ann. Oncol.*, **5,** 901-907.

VOLPE DA, DU DL, ZURLO MG, MONGELLI N AND MURPHY MJ. (1992). Comparative in vitro myelotoxicity of FCE 24517, a distamycin derivative, to human canine and murine hematopoietic progenitor cells. *Invest. New Drugs*, **10,** 225-261.